# Endocardite aiguë isolée de la valve pulmonaire

**DOI:** 10.11604/pamj.2016.25.209.10550

**Published:** 2016-12-06

**Authors:** Amine Tarmiz, Imene Mgarrech, Mehdi Slim, Najeh Ben Hlima, Chokri Kortas, Sofiane Jerbi

**Affiliations:** 1 Service de Chirurgie Cardiovasculaire et Thoracique, CHU Sahloul, Sousse, Tunisie; 2 Service de Cardiologie, CHU Sahloul, Sousse, Tunisie; 3 Service de Cardiologie, CHU Ibn El Jazzar, Kairouan, Tunisie

**Keywords:** Endocardite, valve pulmonaire, chirurgie cardiaque, embolie pulmonaire, Endocarditis, pulmonary valve, cardiac surgery, pulmonary embolism

## Abstract

L'endocardite du cœur droit touche dans la plupart des cas la valve tricuspide, notamment chez les toxicomanes. L'infection isolée de la valve pulmonaire est rarement retrouvée. Nous rapportons dans cette observation le cas d'une jeune femme âgée de 32 ans chez quile diagnostic d'endocardite communautaire de la valve pulmonaire a été confirmé. Cette observation est d'autant plus intéressante qu'elle survient chez une patiente sans antécédents de toxicomanie, avec néanmoins la notion de communication interventriculaire restrictive. La patiente a été opérée en urgence devant l'image de grosse végétation très mobile sur les données de l'échographie cardiaque transthoracique. La chirurgie a été pratiquée sous circulation extracorporelle et la valve pulmonaire a été remplacée par une bioprothèse. Les hémocultures et la culture de la valve ont retrouvé un staphylococcus aureus sensible à la Méticilline. L'évolution postopératoire a été favorable avec un recul de 06 mois sans récurrence infectieuse.

## Abstract (en)

Right heart endocarditis mainly affects tricuspid valve, especially in drug users. Isolated pulmonary valve infection is rare. We here report the case of a 32-year old young woman whose diagnosis of community-acquired pulmonary valve endocarditis was confirmed. This study is even more interesting because pulmonary valve endocarditis has occurred in a patient without a history of drug addiction; nevertheless, it wasn’t associated with restrictive interventricular communication. The patient underwent emergency surgery due to the visualization of large and mobile vegetation on transthoracic heart ultrasound. Surgery was carried out under extracorporeal circulation and pulmonary valve has been replaced with a bioprosthesis. Bloodcultures and valve culture showed methicillin-sensitive staphylococcus aureus. Postoperative evolution was favorable, with a follow-up of 06 months without recurring infections.

## Introduction

L´endocardite du cœur droit touche quasi-exclusivement la valve tricuspide. Nous rapportons dans cette observation un cas exceptionnel d´endocardite isolée de la valve pulmonaire opérée à la phase aiguë.

## Patient et observation

Une jeune femme âgée de 32 ans est hospitalisée en Maladies Infectieuses pour exploration d´un purpura fébrile évoluant depuis 4 jours. À part une communication interventriculaire (CIV) restrictive diagnostiquée au jeune âge, la patiente ne rapporte aucun antécédent pertinent. Elle ne déclare aucune notion de toxicomanie. Néanmoins, on note la notion de panaris du pouce droit deux semaines auparavant.

À l´examen physique, les constantes vitales sont normales, mais la patiente est fébrile à 38,8°C avec un purpura généralisé. L´auscultation cardiaque met en évidence un souffle systolique d´intensité 3/6 au foyer pulmonaire, irradiant à tous les autres foyers. Le reste de l´examen physique est sans particularités. L´électrocardiogramme montre un rythme régulier sinusal à 105 battements/minute sans troubles de repolarisation ni de conduction. La radiographie du thorax est sans anomalies.

L´échocardiographie transoesophagienne retrouve une CIV périmembraneuse restrictive de 4 mm avec une grosse végétation très mobile, de 29 mm de diamètre, sur le versant ventriculaire de la valve pulmonaire, s´attachant à cette dernière par un pédicule très fin. Par ailleurs, la fonction biventriculaire ainsi que les pressions pulmonaires sont normales. Il existe par ailleurs une insuffisance tricuspidienne 1/4.

Le bilan biologique initial retrouve une hyperleucocytose à 18 000 leucocytes/mm^3^ et une CRP à 216 mg/l. Une série de 2 hémocultures successives sont revenues positives au staphylococcus aureus méticillino-sensible. L´indication opératoire est posée en urgence devant le risque embolique majeur.

**Figure 1:**
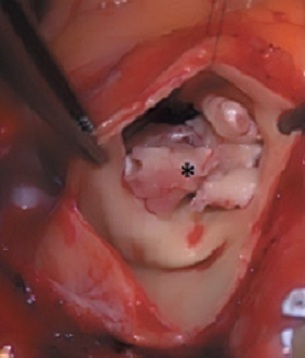
Vue per-opératoire montrant, à travers une artériotomie pulmonaire, une grosse végétation (astérisque) sur la valve pulmonaire

La chirurgie est réalisée sous circulation extracorporelle conventionnelle en normothermie, avec clampage aortique et cardioplégie au sang chaud et riche en potassium. La valve pulmonaire est abordée par une artériotomie longitudinale du tronc de l´artère pulmonaire. L´exploration montre une valve pulmonaire dysplasique et partiellement délabrée par le processus infectieux, avec une grosse végétation de 3 cm appendue au versant ventriculaire de la sigmoïde postérieure de cette valve (Figure 1). On procède à une résection de toute la valve pulmonaire, emportant la végétation. La CIV est vue par cette voie mais son abord est jugé difficile. L´artériotomie pulmonaire est alors complétée par une atriotomie droite.

La valve tricuspide est indemne de lésions. La CIV est périmembraneuse, à extension infundibulaire, faisant environ 6 mm de taille. Elle est fermée par interposition d´un patch de péricarde autologue. Ensuite, une bioprothèse n°27 est mise en place en position pulmonaire via l´artériotomie.

La patiente est mise sous Oxacilline (12g/24h pendant 4 semaines) et Gentamicine (3mg/Kg/24h pendant 5 jours). La culture de la végétation a confirmé la présence de staphylocoque doré méticillino-sensible. L´évolution ultérieure est favorable. Le suivi à 6 mois ne montre pas de récurrence infectieuse.

## Discussion

Nous rapportons ainsi une observation d’endocardite de la valve pulmonaire, dans un contexte de septicémie à staphylocoque doré secondaire vraisemblablement à une infection cutanée, sans aucune notion de toxicomanie. L’intérêt de cette observation réside dans la rareté de l’atteinte endocarditique isolée de la valve pulmonaire. En effet, l’atteinte valvulaire pulmonaire est le plus souvent associée à une atteinte tricuspide et exceptionnellement isolée, représentant tous cas confondus moins de 2 % des endocardites infectieuses [1]. Les endocardites du cœur droit représentent environ 5 à 10% des endocardites infectieuses [1]. Il a été estimé que plus de 75% des endocardites des consommateurs de substances intraveineuses impliquaient le cœur droit, alors que cette localisation ne concernait que 9% des endocardites des patientsnon toxicomanes [2]. La plupart des cas décrits concernent des enfants avec des malformations congénitales, des toxicomanes, des patients porteurs de cathéters intraveineux, voire de cathéters dans l’artère pulmonaire.

Sur le plan bactériologique, dans 50 % des cas le germe retrouvé est un staphylocoque, essentiellement un staphylocoque doré [3]. Selon Hamza [1], les facteurs prédisposant sont dans 30 % des cas une prise répétée de substances par voie intraveineuse, dans 14 % des cas un cathéter central, incluant les cathéters de Swan-Ganz, et dans 11 % des cas un éthylisme chronique.

En l´absence de facteurs prédisposants et de cardiopathie sous-jacente connue, le diagnostic reste difficile. Les manifestations peuvent être pourtant bruyantes, avec des accidents emboliques pulmonaires dans au moins 40 % des cas [1, 4]. La sensibilité de l´échographie transthoracique n´est pas fameuse et le recours à l´échographie transoesophagienne doit être la règle en cas de suspicion d´endocardite sur valve pulmonaire. L’évolution est défavorable dans 20 % des cas avec une intervention chirurgicale pour 30 % des patients, dans un contexte d’infection persistante plus souvent que d’instabilité hémodynamique [1].

## Conclusion

L'endocardite isolée de la valve pulmonaire est très rare. Elle a une présentation clinique d’endocardite du cœur droit, avec un potentiel embolique septique pulmonaire; il faut savoir y penser devant un tableau évocateur et une absence de lésions évidentes tricuspides à l’échographie cardiaque.
